# Correlation of PD-L1 expression on tumour cells between diagnostic biopsies and surgical specimens of lung cancer in real life with respect to biopsy techniques and neoadjuvant treatment

**DOI:** 10.1007/s00432-022-04080-4

**Published:** 2022-06-16

**Authors:** D. Gompelmann, K. Sinn, J. Brugger, D. Bernitzky, B. Mosleh, H. Prosch, S. Geleff, A. Blessing, A. Tiefenbacher, K. Hoetzenecker, M. Idzko, M. A. Hoda

**Affiliations:** 1 Division of Pulmonology, Department of Internal Medicine II, Medical University Vienna, Währinger Gürtel 18-20, 1090 Vienna, Austria; 2 Department of Thoracic Surgery, Medical University Vienna, Vienna, Austria; 3 Department for Medical Statistics, Informatics and Intelligent Systems, Medical University of Vienna, Vienna, Austria; 4 Department for Biomedical Imaging and Image-Guided Therapy, Medical University Vienna, Vienna, Austria; 5 Department of Pathology, Medical University Vienna, Vienna, Austria

**Keywords:** Lung cancer, PD-L1 expression, Immunotherapy, Biopsy

## Abstract

**Purposes:**

Programmed death-ligand 1 (PD-L1) testing is performed mainly on biopsy specimens in patients with advanced lung cancer. It is questionable whether the small amount of tissue analysed in biopsies may represent the true PD-L1 expression of a tumour.

**Methods:**

In this retrospective study, PD-L1 expression on tumour cells derived from bronchoscopy brush cytology, endobronchial ultrasound guided transbronchial needle aspiration (EBUS-TBNA), endobronchial biopsy, transbronchial biopsy (TBB) and computed tomography (CT)-guided transthoracic biopsy was compared to the PD-L1 expression of the corresponding surgical resection in lung cancer patients with regard to neoadjuvant treatment in-between.

**Results:**

A quantitative comparison between the diagnostic biopsy of the primary tumour with corresponding resected surgical specimens in a total of 113 lung cancer patients (60% male, mean age 65 ± 9 years) revealed a statistically significant correlation of PD-L1 expression on tumour cells (*r* = 0.58, *p*< 0.001), for patients without neoadjuvant treatment in-between and for patients who underwent neoadjuvant treatment (both *p* < 0.001). Using a cut-off value of ≥ 50% PD-L1 TPS for comparing the biopsy samples and resected specimens, the concordance rate was 78% with a Cohen’s Kappa of 0.45.

**Conclusion:**

A statistically significant concordance for PD-L1 expression on tumour cells between biopsies from primary lung tumour and resected specimen was found, but of uncertain clinical accuracy. The use of a cut-off value of ≥ 50% PD-L1 TPS resulted only in a moderate agreement. Therefore, the interpretation of the PD-L1 determined form biopsy specimens status should only be considered with caution for treatment decisionsQuery.

## Introduction

Lung cancer is the leading cause of cancer death worldwide among both men and women. The prognosis is mostly determined by disease stage and treatment. Due to newer therapeutic modalities, particularly the molecular targeted therapy and immunotherapy, survival rates have improved in the recent years (Howlader et al. 2020).

For responsiveness to immunotherapy, the expression of programmed death-ligand 1 (PD-L1) on tumour cells was found to be predictive for immunotherapy efficacy (Herbst et al. 2016; Gandini et al. 2016; Xia et al. 2019; Fehrenbacher et al. 2016; Gandhi et al. 2018). The greatest benefit in first-line treatment is observed in patients with advanced NSCLC who have PD‐L1 expression on ≥ 50% of tumour cells (Fehrenbacher et al. 2016; Gandhi et al. 2018). Therefore, PD-L1 testing seems to play a significant role in guiding therapy. Although it must be noted that the results of the various trials, which evaluated predictors for immunotherapy, are controversial, and the association with treatment response varies among different immune checkpoint inhibitors (ICI) (Brahmer et al. 2015).

As a large proportion of patients with lung cancer present with inoperable status at the time of diagnosis, PD-L1 testing is performed mainly on biopsy specimens and not on surgical specimens. However, it is questionable whether the small amount of tissue analysed in biopsies may represent the PD-L1 expression, as the majority of tumours demonstrate a considerable intra-tumoural heterogeneity in PD-L1 expression (Haragan et al. 2019; McLaughlin et al. 2016). Therefore, the question of the reliability of their evaluation in biopsies as compared with corresponding resected surgical specimens was raised. Various trials comparing PD-L1 expression in biopsy and matched surgical specimens revealed controversial results (Ilie et al. 2016; Li et al. 2017; Kitazono et al. 2015; Heymann et al. 2017; Tsunoda et al. 2019). Some authors described a poor correlation of PD-L1 expression on tumour cells between biopsy and corresponding surgical specimens (Haragan et al. 2019; Ilie et al. 2016), whereas other studies found a good concordance of PD-L1 testing between biopsy samples and matches resected specimens (Kitazono et al. 2015; Heymann et al. 2017; Tsunoda et al. 2019; Zhao et al. 2021).

In these trials however, it remains unclear whether the biopsy specimens were derived from metastatic lymph nodes instead of primary lung tumours and whether the patients received a neoadjuvant treatment between the biopsy and surgical resection. These uncertainties and controversial statements may lead clinicians to doubt the suitability of small biopsy specimens for PD-L1 testing.

Moreover, it must be noted that PD-L1 expression on tumour cells may vary due to chemotherapy or immunotherapy which was performed between the initial biopsy and the surgical resection. Clinical trials described a downregulation of the PD-L1 expression of tumour cells after a cisplatin-gemcitabine combination, paclitaxel-based regimen or TKI (tyrosine kinase inhibitor)-based therapy (Sheng et al. 2016; Rojkó et al. 2018). However, in these trials, only bronchoscopy samples were compared to surgical specimens so that a possible impact of other sample technique was not taken into account.

Based on these controversial and still unclear results, the goal of this study was to compare the PD-L1 expression on tumour cells of specimens acquired by various diagnostic biopsy techniques and surgical specimens with consideration of the neoadjuvant therapy.

## Methods

The primary endpoint of this retrospective study was the comparison of PD-L1 expression on tumour cells in biopsy specimen of the primary tumour and corresponding surgical specimen with respect to the biopsy technique and the neoadjuvant treatment that was performed in a subset of this patient cohort.

Patients with the diagnosis of lung cancer assessed by biopsy who underwent surgical resection in 2019–2020 at the Medical University of Vienna were enrolled in this retrospective study. The database queried for this study included the pathological, bronchoscopic, radiological, oncological and surgical reports. The ethics committee of the Medical University of Vienna approved the protocol of this study (1071/2021).

### Subjects and diagnostic procedures

Patients with suspicious pulmonary lesions underwent bronchoscopy or computed tomography (CT)-guided biopsy for establishing histological diagnosis. Thereby, the bronchoscopy techniques included endobronchial biopsy, endobronchial ultrasound guided transbronchial needle aspiration (EBUS-TBNA), transbronchial biopsy (TBB) using forceps and/or brush cytology. Different techniques were combined in several patients for histological diagnosis and/or mediastinal staging. For the comparison of the histology and PD-L1 expression between diagnostic biopsy and surgical regimen, only the PD-L1 expression on tumour cells of the primary tumour but not of lymph node metastases or distant metastases was used. Patients of whom the PD-L1 testing was only available from metastases were excluded from this study.

### Evaluation of PD-L1 expression on tumour cells

Pathological reports were collected for histology and evaluation of PD-L1 expression on tumour cells in biopsy and surgical specimens. All specimens were assessed and scored by experienced pathologists as part of routine diagnostic workup. The histology of the diagnostic biopsies was assessed in 10 different pathology departments using different antibodies for PD-L1 staining, whereas the pathological workup of the surgical specimens was performed only at the Medical University of Vienna. The assessment of PD-L1 expression was only performed on fresh biopsies. PD-L1 tumour proportion score (TPS) was determined. Based on the PD-L1 expression on tumour cells (TPS) patients were divided in 2 subgroups: PD-L1 expression on ≥ 50% of tumour cells and < 50% of tumour cells.

### Statistical analysis

To assess the agreement between PD-L1 measurements of the biopsy and surgical specimen spearman correlation coefficients were calculated. To investigate if the correlation depends on the therapy that patients underwent between biopsy and surgery, scatterplots were drawn with regard to different approaches in preoperative therapy. Spearman correlation coefficients between PD-L1 measurements were calculated separately for patients undergoing chemotherapy, possibly in combination with another form of treatment, and patients without additional therapy between biopsy and surgical resection.

To compare results of the number of times PD-L1 concentrations exceeded the critical bound ( ≥ 50%) a fourfold table was created and Cohen’s Kappa was calculated to assess the agreement between measurements of biopsy and corresponding surgical resection. Spearman correlation coefficients and, in case enough patients were available, their 95%-confidence intervals were computed for subpopulations to investigate whether the correlation is different in certain subgroups. Significance level was set to *α* = 0.05 for all tests, however, no correction for multiple testing was done, therefore *p* values are of descriptive hypothesis-generating character. Statistical analyses were performed using *R*, version 3.6.1 or higher.

## Results

In this retrospective trial, a total of 113 patients (60% male, mean age 65 ± 9 years) with lung cancer of whom PD-L1 expression on tumour cells were available in biopsy specimens and surgical specimens were enrolled in this study. Patients characteristis are presented in Table 1.

For establishing the diagnosis, CT-guided biopsy or bronchoscopy was performed in 36% (41/113) and in 64% (72/113) of patients, respectively. CT-guided biopsies were performed in cutting needle technique using an 18-gauge cutting needle and a 17-gauge coaxial needle. The bronchoscopy techniques included endobronchial biopsy (*n* = 29), EBUS-TBNA (*n* = 12), TBB (*n* = 33) and/or brush cytology (*n* = 31).

The histological workup of biopsy specimens are presented in Table 1. Most of patients had an adenocarcinoma (60.5%) followed by squamous cell carcinoma (31.6%). One patient experienced synchronous carcinoma: a squamous cell carcinoma in the left upper lobe and an adenocarcinoma in the right upper lobe resulting in a total of 114 histological results in 113 patients.

**Table 1:** Patient characteristics, biopsy techniques, histology, PD-L1 expression and neoadjuvant therapeutic regimen

	Study cohort
Patient characteristics	*n* = 113
Male (%, *n*)	60% (71)
Age	65 ± 9
Biopsy techniques	*n* = 146
Brush cytology (%, *n*)	21.2% (31)
Endobronchial biopsy (%, *n)*	19.9% (29)
EBUS-TBNA (%, *n*)	8.2% (12)
TBFB (%, *n*)	22.6% (33)
CT-guided biopsy (%, *n*)	28.1% (41)
Histology derived from biopsy	*n* = 114
Adenocarcinoma (%, *n*)	60.5% (69)
Squamous cell carcinoma (%, *n*)	31.6% (36)
Adenosquamous carcinoma (%, *n*)	3.5% (4)
Large cell/neuroendocrine carcinoma (%, *n*)	0.9% (1)
Carcinoid	0.9% (1)
NSCLC NOS	2.6% (3)
PD-L1 expression on tumour cells derived from biopsies specimens	*n* = 114
Δ TPS (%)	27.9 ± 32.6%
TPS ≥ 50% (%, *n*)	68.4% (78)
TPS < 50% (%, *n*)	31.6% (36)
Neoadjuvant therapeutic regimen	*n* = 27
Chemotherapy alone	74.1% (20)
Chemotherapy and immunotherapy	14.8% (4)
Chemotherapy and radiation therapy	11.1% (3)

In 104 out of 114 (91.2%) matched histological pairs of biopsy and surgical specimen, a similar histology diagnosis was established, whereas in 10 out of 114 (8.8%) matched histological pairs, a change of histology diagnosis was observed: adenosquamous cell carcinoma to squamous cell carcinoma in 2 patients and to adenocarcinoma in 2 patients; NOS (not otherwise specified) to adenocarcinoma in 2 patients and to squamous cell carcinoma in 1 patients; large cell carcinoma to combined large cell carcinoma and squamous cell carcinoma in 1 patient; adenocarcinoma to squamous cell carcinoma in 1 patient and to adenosquamos cell carcinoma in 1 patient.

The median time interval between biopsy and surgery was 56 days [41–100]. In 24% (27/113) of the cases, patients underwent neoadjuvant treatment: chemotherapy alone (*n* = 20), chemotherapy plus immunotherapy (*n* = 4), chemotherapy plus radiation therapy (*n* = 3). Chemotherapy consisted of cisplatin-based regimen or carboplatin-based regimen in 13 and 14 patients, respectively. For immunotherapy, 3 patients received pembrolizumab and 1 patient nivolumab.

### Comparison of PD-L1 expression between biopsy and surgical specimen

**Fig. 1:**
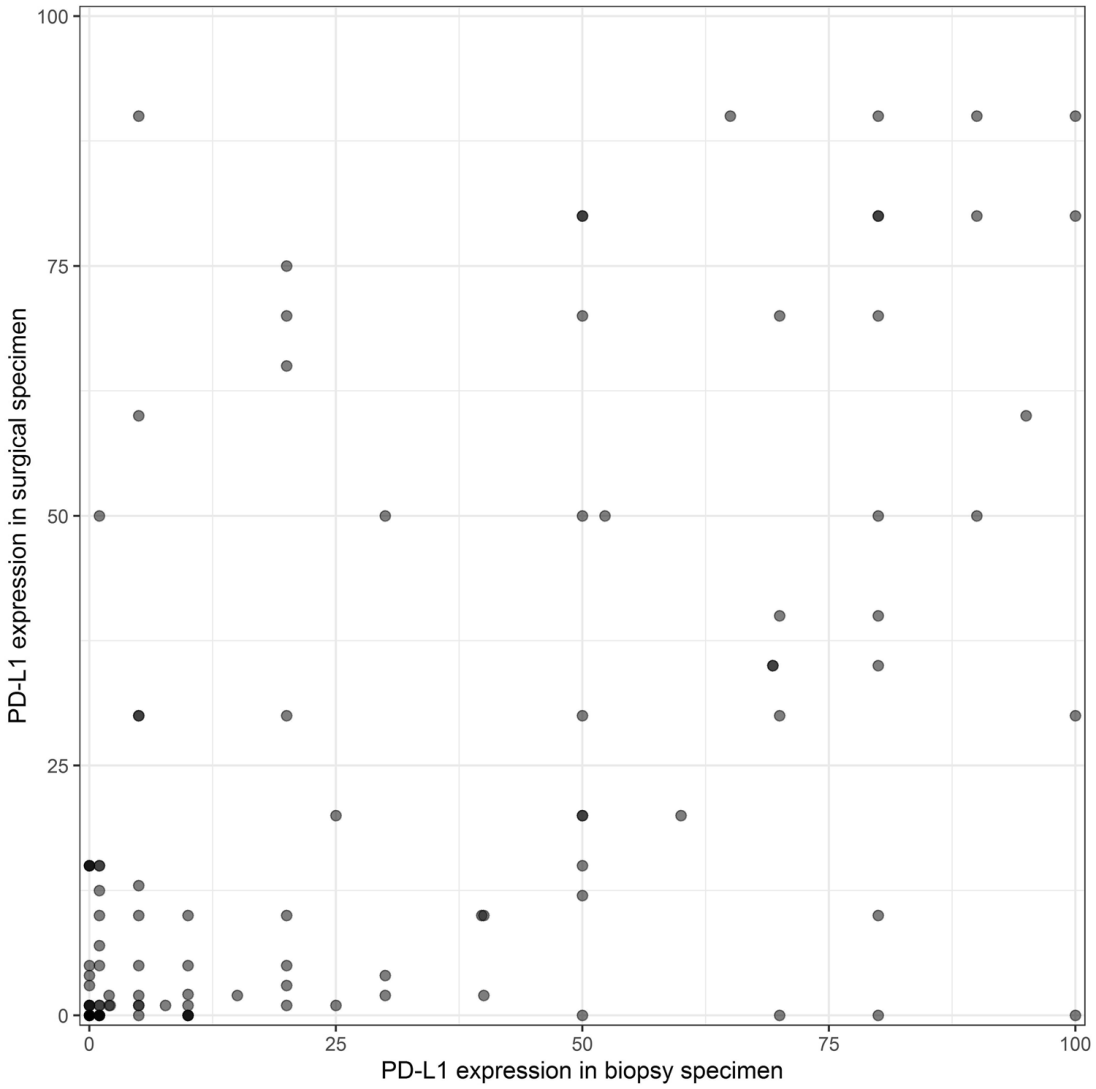
Correlation of PD-L1 testing between biopsies and surgical specimens

PD-L1 testing in 114 biopsy specimens revealed a mean expression of 27.9 ± 32.6% and in surgical specimens 21.9 ± 28.6% on tumour cells. Quantitative comparison between the diagnostic biopsy of the primary tumour with the corresponding resected surgical specimen revealed a significant correlation (*r* = 0.58) of PD-L1 expression on tumour cells (*p* < 0.001). Results are demonstrated in Fig. 1.

TPS was found to be ≥ 50% and < 50% in 68% (78/114) and 32% (36/114) diagnostic biopsies, respectively, and 22% (25/114) and 78% (89/114) in surgical specimens, respectively. In 89 cases, there was a concordance in PD-L1 testing with a cut-off value of ≥ 50% between biopsy and surgical specimen resulting in a concordance rate of 78%. In 25 out of 114 (21.9%) cases there was a discordance. The overall agreement was found to moderate with a Cohen’s Kappa of 0.45.

The comparison of PD-L1 expression between biopsy specimens with corresponding surgical specimens that were both only assessed in one pathology department (*n* = 41) using always the same antibodies (BioSite, Klon BSR90) for staging revealed similar results. Quantitative comparison between the biopsy and resected surgical specimen revealed a significant correlation (*r* = 0.68) of PD-L1 expression on tumour cells (*p* < 0.001). Using cut-off value of ≥ 50% PD-L1 TPS, a fair agreement with a Cohen’s Kappa of 0.38 was found.

### Impact of biopsy tool

In a sub-analysis, the impact of the biopsy tool on the concordance with the PD-L1 testing of the resected tumour was investigated. For brush cytology, endobronchial biopsy, EBUS-TBNA and CT-guided transthoracic biopsy, a statistically significant correlation of PD-L1 expression on tumour cells between the biopsies and surgical specimens were found (Table 2), whereby a superior correlation was found for endobronchial biopsies. Only for the TBB, there was no significant correlation of PD-L1 status between surgically resected specimens and matched biopsy specimens.

**Table 2:** Correlation of PD-L1 testing between biopsies and surgical specimens with regard to biopsy technique

Biopsy technique	*n*	Correlation of PD-L1 TPS values (95% CI)
Brush cytology	31	0.529 [0.241; 0.744)
Endobronchial biopsy	29	0.793 [0.6; 0.898]
EBUS-TBNA	12	0.786 [0.387; 0.937]
TBB	33	0.308 [−0.04; 0.589]
CT-guided transthoracic biopsy	41	0.612 [0.375; 0.774]

*EBUS-TBNA* endobronchial ultrasound guided transbronchial needle aspiration, *TBB* transbronchial biopsy

### Impact of neoadjuvant treatment

**Fig. 2:**
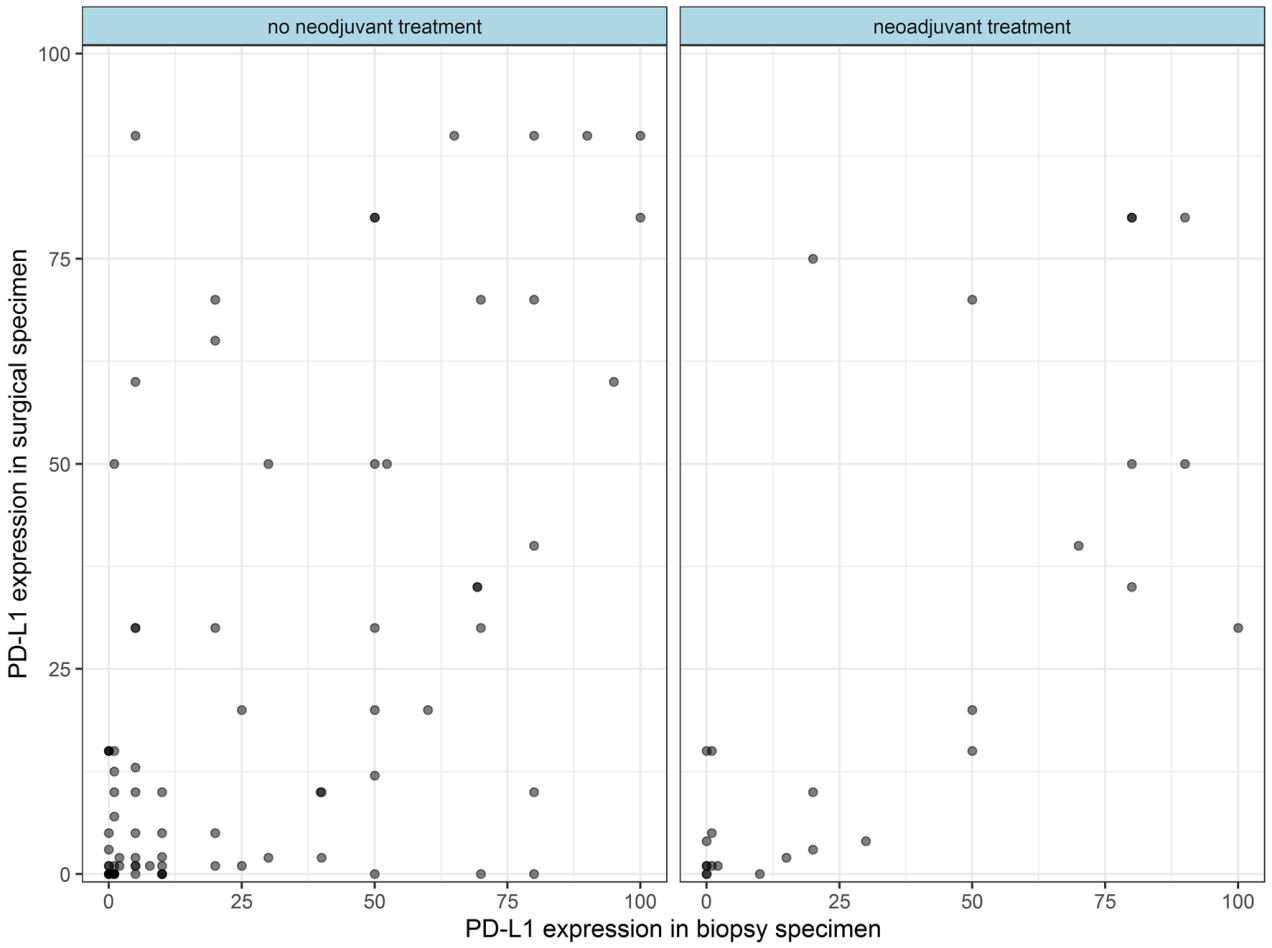
Scatterplots demonstrating the correlation of PD-L1 expression between biopsy and surgical specimens in patients with and without neoadjuvant treatment in-between

Correlation of PD-L1-expression on tumour cells between biopsy and surgical specimens in the subgroup of patients undergoing neoadjuvant treatment was 0.74 (*p* < 0.001). For patients who did not receive neoadjuvant treatment, the correlation was 0.61 (*p* < 0.001). Scatterplots demonstrate the correlation regarding neoadjuvant treatment (Fig. 2).

## Discussion

Immunotherapy improves the survival in patients with advanced lung cancer. Data suggest that a high expression of PD-L1 on tumour cells is associated with a superior response to treatment with ICI (Herbst et al. 2016; Gandini et al. 2016; Xia et al. 2019; Fehrenbacher et al. 2016; Gandhi et al. 2018). Therefore, evaluation of PD-L1 expression on tumour cells may help to select patients who will benefit from immunotherapy providing an individualized therapeutic approach. Furthermore, PD-L1 expression is correlated with major driver and suppressor gene alterations and seems to predict survival in lung cancer patients (Jin et al. 2022). 

As most patients with lung cancer present with advanced inoperable disease at the time of diagnosis, PD-L1 testing is performed mainly on biopsy specimens. However, the heterogeneity of PD-L1 expression within tumours, interassay variability and interobserver disagreement may complicate objective determination of PD-L1 expression on tumour cells particularly in small biopsies (Haragan et al. 2019; McLaughlin et al. 2016). Therefore, our study examined whether the small amount of tissue analysed in biopsies may represent the PD-L1 expression of the tumour depending on biopsy technique and neoadjuvant treatment.

Our results indicated a statistically significant but not necessarily clinically relevant correlation between PD-L1 expression on the tumour cells of diagnostic biopsies of the primary tumour with corresponding resected surgical specimens. This is particularly evident when looking at the comparison using a cut-off of ≥ 50% PD-L1 TPS that resulted only in a moderate agreement. Focussing on biopsy technique, a superior concordance for PD-L1 testing was observed for endobronchial biopsies, an inferior concordance for TBB.

Various studies comparing PD-L1 status of biopsy and surgical specimen resulted in controversial results. Kitazono and colleagues who compared PD-L1 expression between biopsy from the primary tumour and matched surgical specimens in 79 lung cancer patients, confirmed a significant association of the PD-L1 expression in tumour cells between biopsies and resected tumours (Kitazono et al. 2015). A concordance rate of PD-L1 status was found to be 92.4% that is superior to our reported concordance rate of 78%. However, while Kitazono et al. used a hybrid score to describe PD-L1 expression on tumour cells and differed between PD-L1 positivity and PD-L1 negativity, we used a cut-off value of 50% PD-L1 TPS. It must be noted that the interpretation of PD-L1 staining is a challenge for pathologists. PD-L1 immunohistochemistry using a cut-off value is considered to be more challenging than a simple positive or negative result.

In another study which compared PD-L1 expression on tumour cells in different cytology, biopsy and surgical specimens of primary tumours or metastases (lymph node metastases, distant metastases, pleural effusions) in 23 patients independent of treatment in-between, no significant difference between the percentage of PD-L1 positive cytology and histology specimens was found suggesting a good concordance. However, this result was somehow surprising and contrary to findings by Xu et al. who described a poor correlation for PD-L1 expression between primary tumours and metastatic lymph nodes in 76 NSCLC patients (Xu et al. 2019). In our study, only biopsy specimens from primary tumours were compared to corresponding resected tumours.

In contrast to our finding, other studies revealed a poor concordance for PD-L1 expression between biopsy and surgical specimen. Ilie and colleagues who used a scoring system from 0 to 3 depending on PD-L1 expression ( ≥ 50%, ≥ 5% and < 50%, ≥ 1% and < 5%, < 1%) reported a poor correlation between the PD-L1 expression assessed in biopsy specimens and the corresponding resected tumour in 160 patients with NSCLC (Ilie et al. 2016). In this study, biopsy samples were derived from bronchoscopy including EBUS-TBNA or CT-guided transthoracic biopsy, but it remains unclear, whether the biopsy was sampled from the primary tumour or lymph node metastases. Also Li et al. found only a moderate correlation of PD-L1 expression between whole sections from NSCLCs and the corresponding tissue microarrays (TMAs) serving as surrogate biopsy specimens (Li et al. 2017). Of note, in this study, no real biopsy specimens but TMA were used for comparison.

Previously, different studies reported that a cisplatin-gemcitabine combination, paclitaxel-based regimen or TKI-based therapy led to a downregulation of the PD-L1 expression of tumour cells (Sheng et al. 2016; Rojkó et al.^ 2018^). In our study, however, a statistical significant correlation of PD-L1 expression between biopsy and surgical specimen was not only found in treatment-naïve surgical patients, who did not undergo a treatment in-between but also in patients who received neoadjuvant therapy.

The limitation of this study is its retrospective character and the relatively small sample size. Particularly the number of patients who underwent neoadjuvant treatment between the biopsy and the surgical resection is low and does not allow a general statement about concordance of PD-L1 expression between biopsy and surgical samples in this subgroup. Another limitation is, that the histological workup of the biopsy specimen was performed in different pathology units that used different antibodies for staining (BioSite, Klon BSR90, SP263, QR1, Biocare CAL10 and 22C3 pharmDx). However, a good agreement rate in interpretation of the PD-L1 expression using various antibodies is described (Büttner et al. 2017). Moreover, similar results were obtained when regarding only at PD-L1 testing of biopsy and surgical specimens using the same antibody in one pathological department. Therefore, the use of various antibodies for staining in different pathology departments seem not to have substantial impact. It must be noted, that our study in comparison to the other study reflects the real-life situation. The demonstration of a statistically significant correlation in this study is even more significant because it is known that interpretation of PD-L1 expression does have its peculiarities with varying interobserver agreement (Butter et al. 2022).

Summarizing, this study found a statistically significant correlation for PD-L1 expression on tumour cells between biopsy and surgical specimen, but of uncertain clinical relevance. Particular the use of a cut-off value of ≥ 50% PD-L1 TPS resulted only in a moderate agreement. Therefore, the interpretation of biopsy based PD-L1 status should be considered with caution when deciding therapeutic approach for a patient.

## Data Availability

The datasets generated during and/or analysed during the current study are available from the corresponding author on reasonable request.

## Abbreviations

EBUS-TBNA: Endobronchial-ultrasound guided transbronchial needle aspiration
ICI: Immune checkpoint inhibitor
NSCLC: Non-small cell lung cancer
PD-L1: Programmed death-ligand 1
TBB: Transbronchial biopsy
TKI: Tyrosine kinase inhibitor
TMA: Tissue microarrays
TPS: Tumour proportion score
